# Molecular characterization of avian reovirus isolates in Tunisia

**DOI:** 10.1186/1743-422X-10-12

**Published:** 2013-01-05

**Authors:** Ymene Hellal Kort, Hager Bourogâa, Latifa Gribaa, Daniel Scott-Algara, Abdeljelil Ghram

**Affiliations:** 1 Laboratory of Epidemiology and Veterinary Microbiology, Institut Pasteur de Tunis, University of Tunis - El Manar, 13 place Pasteur, BP 74, 1002, Tunis-Belvedere, Tunisia; 2 Unité de Régulation des Infections Rétrovirales, Institut Pasteur, 27 Rue Dr. Roux, 75724, Paris, France

**Keywords:** Avian reoviruses, Malabsorption syndrome, Arthritis, σC gene, σB gene, Genotyping

## Abstract

**Background:**

Genotype analyses of avian reoviruses isolated from organ samples collected from chickens with suspicious clinical symptoms, between 1997–2008, was based on sequences for both σC and σB genes and aligned with those published in the Genbank, making it possible to carry out studies of molecular classification and relationships.

**Methods:**

The full length of the known variable protein σC and part of the σB encoding genes, were amplified with RT-PCR, using conserved primers. PCR products were sequenced and the sequences were analyzed and aligned with avian reovirus sequences from the Genbank database.

**Results:**

The sequences of σC-encoding genes of all the isolated strains indicated their close relationship with the American, Chinese and Indian strains. Taking the American strain S1133 as a reference, the two Tunisian isolates 97.1 and 97.2 showed some nucleotide substitutions. For isolate 97.1, the substitution was silent whereas for strain 97.2 the mutation was at the first position of the corresponding codon and induced the substitution of the amino acid encoded. For the σB-encoding gene, the sequences of the Tunisian strains showed mutations at positions two or three of the corresponding codons, inducing substitutions of amino acids at these positions. The phylogenic trees based on σC and σB encoding genes indicated closer relationship between Tunisian, American and Taiwanese isolates of genotype I.

**Conclusion:**

Our study describes the genotype of avian reoviruses that are not yet well characterized genetically. The characterization and classification of these viruses might be significant for understanding the epidemiology of malabsorption syndrome and viral arthritis, and improving our knowledge of the genotype of strains circulating in Tunisian flocks. Furthermore, the study of their variable pathogenicity could be extremely important in the choice of the appropriate vaccine strain to control disease.

## Background

Avian reovirus (ARV) pathogens are found worldwide [1-3] and their molecular characterization, using PCR and nucleotide sequence analysis, have been described [4-6]. Yet, only a limited number of genomic sequences belonging mainly to the S- class, has been published. These sequences have characterized strains isolated from USA, Taiwan, Germany, Netherlands, Australia, Japan and China [7,8]. Recently, σC protein sequences of some Indian strains were submitted to the Genbank.

Particles of ARV present two layers of capsid and 10 segments of double-stranded RNA [9]. Genomic segments can be separated on polyacrylamide gel electrophoresis into three different classes, based on size, named L (large), M (medium) and S (small). They encode for at least 8 structural and four non-structural proteins [10,11]. Amongst the S-class segments of ARV, the segment S1 contains three open reading frames that are translated into P10, P17 and σC proteins [10,11]. The protein P10 induces cell-cell fusion [12,13] while P17 causes cellular protein translation shutoff and cell cycle arrest [14,15]. The σC protein plays a role for virus attachment [16] and as apoptosis inducer [17]. It has been demonstrated that cell entry of avian reovirus follows a caveolin-1-mediated and dynamin-2-dependent endocytic pathways that require activation of p38 MAPK and Src signaling pathways [18]. It has also been suggested that it represents the target for type-specific neutralizing antibodies. The σB protein, encoded by the S3 gene [10,19], carries group-specific neutralizing epitopes [20]. Recently, the ARV σA protein, encoded by the S2 gene [16], has been identified as a double-stranded RNA binding protein that may be involved in interferon resistance [21]. Another viral protein, σNS encoded by the S4 gene, has been reported as having a single-stranded RNA binding activity [22,23].

This paper reports the analyses of the σC and part of the σB encoding genes along with the nucleotide sequences of 15 ARV isolates, identified over a 12-year period. To the best of our knowledge, this is the first report regarding the genotypic classification of ARV in Tunisia and in Africa. Because ARV are important poultry pathogens, causing arthritis, chronic respiratory disease and malabsorption syndrome, which result in considerable economic losses to the poultry industry [24,25], better understanding their pathogenicity is economically important.

## Methods

Being the major laboratory doing diagnostic and research for poultry diseases in Tunisia, samples from different flocks and regions were sent to the laboratory, by private and governmental veterinarians, for the diagnostic of major avian diseases. Fifteen ARV isolates were identified from chickens with viral arthritis, malabsorption syndrome or other suspected symptoms. The flocks were located in governments in the north (Bizerte, Ben Arous and Nabeul), the center (Sousse, Monastir and Mahdia) and the south (Sfax) of the country. ARV were propagated in specific pathogen-free (SPF) embryonnated chicken eggs, followed in cultures of primary chicken embryo fibroblast (CEF) or liver (CEL) cells [7]. The virus was detected when strong cytopathic effects (CPE) were observed after 3 to 5 subcultures; virus stocks were subsequently prepared in 75 cm2 flasks. Once 70-80% CPE were developed, cell cultures were frozen and thawed 3 times, then clarified with low centrifugation at 3000rpm for 20min. Supernatants were then conserved in aliquots at −80°C for later use. The vaccine strain (Nobilis S1133) was propagated in CEF cells and treated as described above. Viral RNA was extracted from supernatants using Trizol (Gibco Brl) as per the procedural modification described by Lee et al. [26]. Briefly, 1ml of Trizol was incubated with 300 μl of clarified supernatant for 5min, at room temperature. RNA was then isolated with chloroform (Sigma-Aldrich, France) and precipitated with isopropan-alcohol (Sigma-Aldrich, France) overnight. Total RNA was used as a template for RT-PCR reactions; non infected-cells and Rnase free water were used as negative controls.

### Primers

Primers used for RT-PCR reactions (Table 1) were selected according to the sequences of S1 and S3 segments from available ARV sequences [4,19,27].

**Table 1:** RT-PCR primer sequences and expected PCR products

**Designation**	**Sequence (5’-3’)**	**Gene**	**Location**^**a**^	**PCR products (bp)**
S1C	ATTGAATTCTCTCTGTTATCTAACCTTG	σC	446-472	738
S1D	AAGGAATTCGTTGAGAACAGAAGTAGG	σC	1183–1157	
S1E	TCTGAATTCATCCGCAGCGAAGAGAGGTG	σC	630–658	342
S1F	AGTGAATTCAGTATCGCCGTGCGCAG	σB	971–943	
S1G	CCTGAATTCGTGACTGACTTAACGAAC		910–936	720
S1I	ACGAATTCTTTCATTAGACATGGACC		1130–1156	520
S1H	TAAGAATTCCCAGTACGGCGCCACA		1629–1603	
P1	TAACATCTAGCTATTTG		67–83	627
P2	CAAGCATTACAGGGCCAGC		720-738	

^a^ Location according to Yin et al.[19] and Liu et al. [27,28].

### Reverse transcription (RT), PCR and double Nested PCR (N-PCR)

A double N-PCR amplified the full length of the σC encoding gene. For the RT reactions, RNA was denatured in the presence of S1C or S1G forward primers (50pmol) and 1 μl RNasin (Promega) in a total volume of 11 μl, for 10 min, and rapidly cooled on ice. Subsequently, 9 μl RT mixture, containing 4 μl 5X first strand buffer (Invitrogen), 2 μl DTT and 2 μl dNTP (10 mM)) were added to each sample. After 2min incubation at 42°C, 1 μl superscript reverse transcriptase (Invitrogen) was added to each tube. The RT was carried out at 42°C for 50 min. The cDNA amplification was performed in 10 μl volume, containing 1,5 mM MgCl_2_, 1X PCR buffer (Invitrogen), 2,5U *Taq* DNA Polymerase (Invitrogen), 0,5 μM of each primer (S1C and S1D, S1G and S1H) and 5 μl of sample from the RT reaction. PCR reactions were subjected to 35 cycles (denaturation for 1 min at 94°C, annealing for 1 min at 55°C, extension for 2 min at 70°C) and one final extension cycle at 70°C for 10 min for the amplification of the whole σC gene. For the N-PCR, 5 μl of 1/10 or 1/100 of each RT-PCR product was amplified using primer sets (S1E and S1F, S1I and S1H) with the same components as for the PCR reaction. Samples were subjected to 35 cycles (45s denaturation at 94°C, 1min annealing at 55°C and 1min extension at 70°C) with a one final extension at 70°C for 7 min.

For the σB gene, samples were treated as described above. RT was performed using P1 primer and samples were subjects to 35 cycles (denaturation for 5 min at 94°C, annealing for 30 s at 50°C and extension for 70s at 70°C) with a one final extension for 7 min at 70°C.

### Analysis of amplified products

After completion of the PCR reactions, 10μl of reaction mixtures were loaded onto a 2% agarose gel for 1 hour in TAE buffer (90 mM Tris–HCl, 90 mM acetic acid, 2 mM EDTA, pH 8,3), containing 5 μl/ml ethidium bromide, for electrophoresis and subsequent visualization with ultraviolet transilluminator. A DNA ladder of 100 bp was run as a size marker.

### Sequencing analysis

PCR products were used for direct sequencing using forward and reverse primers to obtain the full length of σC and part of σB encoding genes. Sequencing was done three times using PCR products of the same isolate to avoid cross contamination. The ABI Prism Big Dye Terminator Cycle Sequencing Reaction kit (Applied Biosystems, Foster City, CA) was used and electrophoreses were run on polyacrylamide gel POP 7 in a four-capillary Applied Biosystem Genetic Analyser. Nucleotide sequences were aligned for comparison using “Clustal W” from Bio-Edit Sequence Alignment Editor [29,30]. The sequences were deposited in the Genbank database (see Table 2 for the accession numbers of the nucleotide sequences).

**Table 2:** Virus used in this study and sequences submitted to Genbank by us and others

**Isolate**	**Symptoms**^**a**^	**Origin**	**Year**^**b**^	**Genbank accession number**^**l**^	
				**σC**	**σB**
S1133	V.A.^c^	USA	1973	AF330703	U20642
2408	Mal.S.^d^	USA	1983	AF204945	AF208038
1733	Mal.S.	USA	1983	AF004857	AF004856
138	V.A.	USA	UN^f^	AF218359	AF059721
176	V.A.	USA	UN	AF218358	AF059720
42563-4	R.S.S^e^	USA	2005	DQ872801.1	N.A.
GA41565	R.S.S.	USA	2005	DQ872799.1	N.A.
GA41560	R.S.S.	USA	2005	DQ872798.1	N.A.
GA 40973	RS.S	USA	2005	DQ872797.1	N.A.
MS46523-1	R.S.S.	USA	2005	ABJ09661.1	N.A.
GA40963	R.S.S.	USA	2005	ABJ09657.1	N.A.
V.A.Vac	Vaccine^g^	USA	2006	EF122837	N.A.
AVS-B	R.S.S.	USA	2005	YP004226527	YP004226529
					JR1	N.A^h^	England	UN	EF122836.1	N.A.
**TU399**	**V.A.**	**Tunisia**	**1998**	**HM751135**	**HM751120**					
**TU430**	**V.A.**	**Tunisia**	**1998**	**HM751137**	**HM751122**					
**TU429**	**Mal.S.**	**Tunisia**	**1998**	**HM751136**	**HM751121**					
**TU435**	**V.A.**	**Tunisia**	**1998**	**HM751138**	**HM751123**					
**TU96**	**V.A.**	**Tunisia**	**1999**	**HM751139**	**HM751124**					
**TU97.1**	**V.A.**	**Tunisia**	**1999**	**HM751140**	**HM751125**					
**TU97.2**	**V.A**	**Tunisia**	**1999**	**HM751141**	**HM751126**					
**TU119**	**Unclear**^i^	**Tunisia**	**1999**	**HM751142**	**HM751127**					
**TU420**	**Unclear**	**Tunisia**	**2000**	**HM751143**	**HM751128**					
**TU87**	**Unclear**	**Tunisia**	**2001**	**HM751144**	**HM751129**					
**TU5**	**Mal.S.**	**Tunisia**	**2002**	**HM751145**	**HM751130**					
**TU71B1**	**Unclear**	**Tunisia**	**2002**	**HM751146**	**HM751131**					
**TU105B6**	**Mal.S.**	**Tunisia**	**2002**	**HM751147**	**HM751132**					
**TU360**	**Mal.S.**	**Tunisia**	**2004**	**HM751148**	**HM751133**					
**TU1390**	**Mal.S.**	**Tunisia**	**2008**	**HM751149**	**HM751134**					
NLI1296M	Mal.S	Netherlands	1996	AF354230.1	N.A.					
NLI0298M	Mal.S	Netherlands	1998	AF354229.1	N.A.					
NLA1396T	V.A.	Netherlands	1996	AF354228.1	N.A.					
99G	Unclear	China	1999	N.A.	DQ415659					
T-98	V.A.	China	2006	ABK51295.1	ABK32526.1					
C-98	V.A.	China	2006	ABK51292.1	ABK32523.1					
G-98	V.A.	China	2006	ABG43119.1	ABK32524.1					
HB06	V.A.	China	2008	ACB11346.1	N.A.					
OS161	Mal.S.	Japan	1970	AF204946	AF301471					
Fahey and Crawley	R.D.^k^	Canada	1954	ABI97289.1	N.A.					
GEL13a98M	Mal.S	Germany	1998	AF354226.1	N.A.					
GEL13b98M	Mal.S	Germany	1998	AF354227.1	N.A.					
GEL12 98M	Mal.S	Germany	1998	AF354225.1	N.A.					
GEL06 97M	Mal.S	Germany	1997	AF354224.1	N.A.					
GEL05 97M	Mal.S	Germany	1997	AF354223.1	N.A.					
GEL03 97T	V.A.	Germany	1997	AF354222.1	N.A.					
GEL01 96T	V.A.	Germany	1997	AF354221.1	N.A.					
GEI09 97M	Mal.S	Germany	1997	AF354220.1	N.A.					
GEI1097M	Mal.S	Germany	1997	AF354219.1	N.A.					
K5	N.A.	India	2008	EU681261.1	N.A.					
820	N.A.	India	2008	EU681260.1	N.A.					
MKA	N.A.	India	2008	EU681259.1	N.A.					
MS-4	N.A.	India	2008	EU681258.1	N.A.					
1884	N.A.	India	2008	EU681257.1	N.A.					
6-10	N.A	India	2008	EU681256.1	N.A.					
VA-1is	V.A	India	1983	EU681254	N.A					
VA-1	Vaccine	India	2008	EU681255.1	N.A.					
Bareilly son-1	N.A.	India	2008	HM015906.1	N.A.					
Bareilly	N.A.	India	2008	FJ949087.1	N.A.					
T6	R.D	Taiwan	1970	AF204948	AF208036					
750505	V.A.	Taiwan	1986	AF204950	AF208035					
919	Normal	Taiwan	1992	AF204949	AF208034					
601G	V.A.	Taiwan	1992	AF297217	AY008384					
601SI	VA.	Taiwan	1992	AF204947	AF208037					
R2/TW	V.A.	Taiwan	1992	AF297213	AF301472					
918	Mal.S.	Taiwan	1992	AF297215	AF301473					
916	Mal.S.	Taiwan	1992	AF297214	AY008383					
1017-1	Mal.S.	Taiwan	1992	AF297216	AF301474					

^a^: Symptoms observed in infected chickens; ^b^: year of isolation of the corresponding strain; ^c^: viral arthritis; ^d^: malabsorption syndrome; ^e^: Runting-stunning syndrome; ^f^: year of isolation not known; ^g^: sequences of vaccines derived from pathogenic strains S1133 or VA-1is; ^h^: data not available; ^i^: chicken's symptoms not described; ^j^: apparent healthy chickens; ^k^: Respiratory disease; ^l^: Accession Numbers submitted by us or according to Shapouri et al. [4]; Kant et al. [7]; Shmulevitz et al. [31]; Liu et al. [27,28]; Zhang et al. [32]; Guo et al. [33].

### Phylogenic analysis

The full length of the σC and only part of the σB gene sequences were translated using the Bio-Edit program. The phylogenetic tree, for either epidemiological or phylogenic relationship studies, was constructed using Splits Tree version 4.10 Software with the Neighbour-Joining (NJ) method and bootstrap analysis (n=1000), to determine the best fitting tree for each gene [34].

## Results

Fifteen Tunisian ARV were isolated (Table 2) in SPF eggs, CEF and/or CEL cell cultures. S1133 vaccine strain, used as a control, was propagated in CEF cultures. Aliquots of clarified freeze-thawed cell supernatants (4 or 5 passages) were used for RNA extraction. Using primer pairs of S1C-S1D, S1E-S1F, S1G-S1H, S1I-S1H for the σC gene and P1-P2 for the σB gene, RNA extracts from all ARV isolates as well as the S1133 strain was transcribed into cDNA and amplified, producing fragments with expected sizes of 738, 342, 720, 500 and 672 bp, respectively. PCR products from S1133 strain and the Tunisian isolates were obtained, indicating that primer regions were conserved in our strains. RNA extracted from non-infected cells and Rnase-free water were used as negative controls and no DNA amplification was observed, indicating that the amplified viral DNA was specific and did not originate from contamination.

### Sequence analyses and phylogenic study

To gather information about the genetic classification of the Tunisian isolates, different sequences of σC and σB encoded genes, from pathogenic and vaccine strains were analysed. ARV sequences are labelled as submitted by our laboratory or by others (Table 2) [4,7,27,28,31-33].

**Figure 1:**
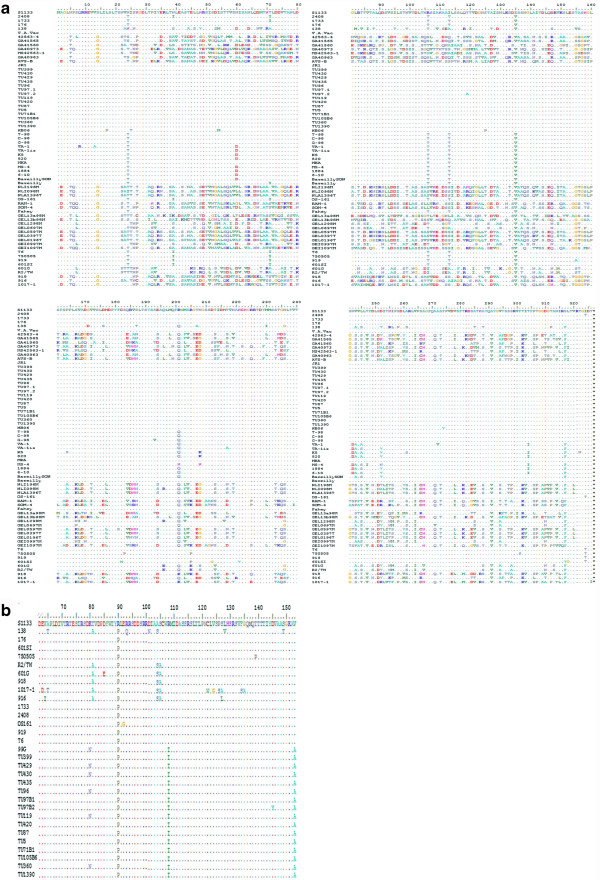
**Multiple sequence alignment of σC (326 AA) (a) and part of σB proteins (b) (residue 67 to 153) of ARV.** The predicted AA sequences of σC and part of σB proteins of 15 Tunisian ARV isolates (TU339, TU429, TU430, TU435, TU96, TU97.1, TU97.2, TU119, TU420, TU87, TU5, TU71B1, TU105B6, TU360 and TU1390) were aligned and compared to previously published sequences using 'Clustal W' from Bio-Edit Sequence Alignment Editor [27,29]. Sequencing was done three times using PCR products of the same isolate to avoid cross contamination. AAs are numbered up the sequences residues. AAs identical to the S1133 pathogenic strain sequence are indicated by dot. Genbank accession numbers of the strains, either determined in this study or submitted by others, are cited in Table 2.

Alignment of nucleotides of the σC encoded gene of all identified strains showed a close relationship with the American S1133 and the English JR1 pathogenic strains [33] (Table 2). Analyses of the nucleotide sequences revealed that the Tunisian strains showed the same sequences as the strain S1133, except for the 97.1 and 97.2 isolates. The first one showed a substitution of Guanine with Adenine at the position 354. This mutation was observed for all Chinese, Japanese and Indian strains (pathogenic and vaccine ones) and the only isolate from Canada. However, six of all American (2048, 1733, 138, 176, GA41560, MS42563-1), seven of all Taiwanese (T6, 70505, 919, 601G, 601SI, R2/TW, 916) and one of two Australian (SOM-4) pathogenic strains showed the same mutation. Moreover, the 97.1 had the same sequence as the only English pathogenic strain JR1, except that the later shows a substitution of Guanine with Thymine at the position 927. Multi-alignment of deduced amino acid (AA) sequences revealed that the mutations at positions 354 and 927 were synonyms; thus no differences were found in the AA sequences between 97.1, S1133 and JR1 strains (Figure 1a). For the second strain 97.2, a substitution of Adenine with Guanine at position 355 of the σC gene was observed. This mutation was at the first position of the corresponding codon and induced the substitution of the amino acid encoded (Figure 1a). Same mutation was likewise detected for the German strain GEL13a98M and the two American strains MS42563-1 and 42563–4. Nevertheless, at this position, three of German (GEL0597M, GEL0397T, GEL0196T) and Dutch (NLA1396, NLI098M, NLI196) strains and two Taiwanese strains (918, 1017–1) showed one Cytosine. The two American strains GA40963 and GA41565, however, showed one Thymine at this position.

Using S1133 as a reference strain, alignment of nucleotide sequences of the σB encoded gene, showed some substitutions. The first one, located at the position 308, was observed only for TU430 and TU360 isolates. The second substitution located at position 438, was observed for strains TU429 and TU430 which were isolated in 1998, for TU96 and TU119, isolated in 1999 and for TU360, collected in 2004. Lastly, three substitutions were observed at positions 467, 521 and 656 for all Tunisian strains. Multi-alignment of these strains with the most published ones indicated that the sequences of their σB encoded gene are closely related to the Chinese 99G strain, which shows the same nucleotide sequence as the three isolates (TU429, TU96, TU119). Deduced AA sequences demonstrated that all the observed mutations are non-synonymous and imply substitutions of the corresponding AA (Figure 1b).

**Figure 2:**
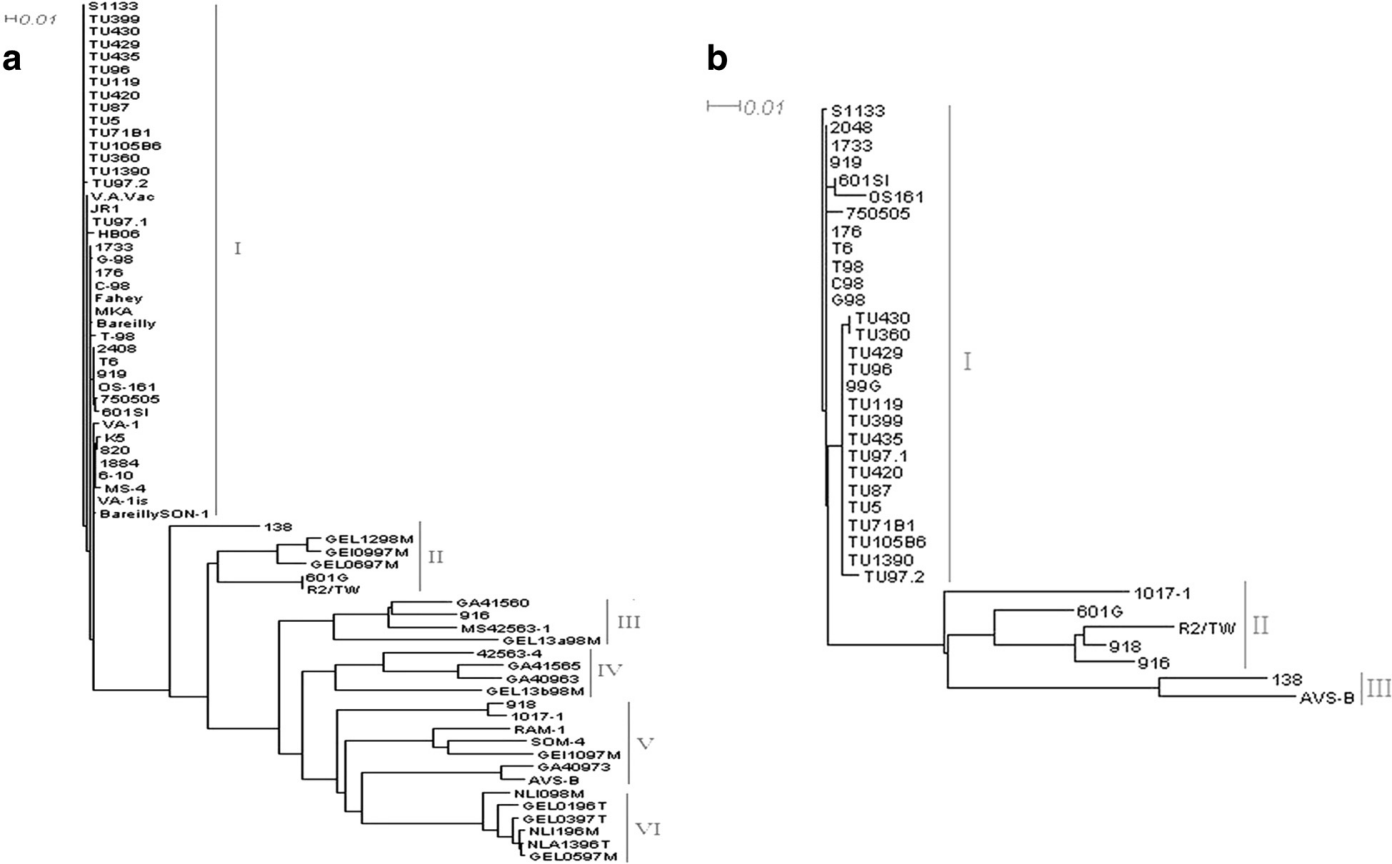
**Phylogenic trees of the σC (a) and part of the σB (b) genes of Tunisian ARV strains.** The trees were generated on the base of nucleotides sequences using splits tree phylogenic software (version 4.10) with distance-based neighbour-joining (NJ). The reliability of the trees was assessed by bootstrap analysis with 1.000 replications; only bootstrap values of > 90% are shown. The length of the horizontal lines is proportional to the minimum number of nucleotide differences required to join nodes. The vertical lines are spacing branches and labels. The scale bar is proportional to the phylogenic distance (Table 2).

Phylogenic comparison of genotype clustering based on nucleotide sequences of the σC gene of the Tunisian isolates with published one, showed that they are closely related to each other and can be classified in cluster I with all Indian and Chinese strains as well as those isolated from England, Japan and Canada. They remain different from Australian and Dutch isolates, found only in the clusters V and VI, respectively. The Taiwanese isolates are more dispersed and evolved in at least 4 clusters. The isolates from Germany and America are the most dispersed ones and grouped in 5 different clusters and only one American strain 138 is placed in cluster II (Table 2, Figure 2a) [7,8,27].

For the σB gene sequences, the phylogenic tree showed that the Tunisian strains are relatively closer to each other and can be classified in the same cluster I with the only Japanese strain OS161, all Chinese and four Taiwanese strains. The remainder Taiwanese strains are classified in cluster II and III, respectively. However, the American strains are dispersed in clusters I and III (Table 2, Figure 2b).

## Discussion

ARV pathogenicity is very heterogeneous and ARV strains are associated with disease conditions such as viral arthritis (VA), tenosynovitis and Malabsorption Syndrome (MAS). They are also isolated from chickens without any clinical signs [35]. Although the relationship between reovirus and tenosynovitis has been established, a causative role is less clear in MAS [36]. The characterization and the classification of ARV might be important for the epidemiology of MAS and VA for the choice of the appropriate vaccine in farms with ARV problems. Although ARV infections still cause problems in poultry, the only available ARV sequences are for strains from the USA, Canada, Taiwan, Australia, the Netherlands, Germany, Japan, England and China. Recently, σC encoded gene sequences from Indian isolates became available in Genbank [7,8] (Table 2).

Genotyping of ARV using σC encoded gene revealed at least 6 different genotypes [27], but correlations between genotypes, pathogenic characteristics and serotype classification have not been well established yet. A phylogenic study using the σC encoded gene indicated that, despite the close relationship with the first USA isolates belonging to clusters I and II, those recently isolated from chickens with Runting-Stuning Syndrome (RSS) showed dispersion and were classified in the 3 different clusters III, IV and VI (Table 2, Figure 2b). They remain quite different from Australian and Dutch strains, classified in the clusters V and VI, respectively. The first strains isolated from Taiwan were classified in cluster I, whereas the more recent ones were dispersed between the 3 clusters II, III, and V. Furthermore, the German strains isolated during the last two years are much more dispersed and classified into 5 clusters. Finally, the trypsin-resistant strain JR1 is classified in the cluster I [8,27,33].

Molecular characterization of the σC sequences of all Tunisian ARV, isolated from different field conditions over a twelve years period and compared to several published strains, allowed their classification in one cluster. However, no correlation could be established between the σC gene sequences and the disease conditions in the field. ARV were also isolated from apparently healthy chickens, making their classification in a true lineage difficult as stated by Kant et al. [7].

The σC-encoding gene has evolved into six clusters, while the other S-class genes have diverged into two to four clusters [27]. It displayed features of the highest level of sequence divergence and rapid evolution. Therefore, this gene could be used as a genetic marker for the classification of ARV isolates [27,28]. Such classification in clusters is independent of determination of the 5' end, the 3' end or the whole ORF of the σC gene. Furthermore, sequencing of a representative part of this gene seems to be sufficient [7].

The major part of σC gene sequences of the Tunisian isolates was closely related to the very well-known American strain S1133. Only nucleotide sequences of the 97.1 and 97.2 strains showed mutations at the positions 354 and 355, respectively. A relationship between the 97.1 and JR1 strains is possible; however, data about the σB gene sequence of the later is not yet published for a possible gene comparison.

Analyses of the whole σB-encoding gene were performed to allow rapid detection of ARV infections with different Tunisian isolates. The constructed Phylogenic trees showed in previous studies that the gene evolves into 2–3 lineages [8,27]. Analyses of the published sequences demonstrated that it evolves into 3 clusters, results obtained when using sequences of the whole gene or the representative sequence used in our work. Recently, we performed a restriction fragment length polymorphism (RFLP) with two restriction enzymes that cut in two sites among those containing mutations on our amplicons (data no shown). PCR/RFLP can be a procedure used for rapid characterization and differentiation of Tunisian ARV isolates.

Mutations do exist in the σB gene sequences of Tunisian strains, allowing their classification into 4 groups: a first group containing the TU430 and TU360 isolates; a second one with the TU429, TU96 and TU119 isolates; a third group containing the TU339, TU87, TU5, TU420, TU105B6, TU1390, TU71B1, TU435 and TU97.1 isolates and the fourth group with only the TU97.2 isolate. The substitution at position 467 was observed for all ARV with respect to the S1133 strain.

Sigma B protein was described as a high variable protein and possesses group-specific neutralizing epitopes [37]. Alignment analysis of amino acids of part of the σB protein showed that only the substitution at position 171 is silent, whereas all other mutations at positions 308, 438, 467, 521 and 656 are non-synonymous and induced substitution of the corresponding translated AA (Figure 1b). These mutations were also observed for the Chinese isolate 99G and indicated a possible relationship between the Tunisian and the Chinese ARVs. Unfortunately, the σC gene sequence of the 99G isolate is not available for comparison. For the Taiwanese strains, the majority of nucleotide substitutions were silent because they occur at the third position of the codons [27].

The σC protein, located on the surface of the outer layer capsid, induces neutralizing antibody production and is the determinant for ARV serotypes [16]. In previous studies, ARV strains were differentiated by virus neutralization assays [38]. Serological classification of ARV strains has not been successful because of high cross-reactivity of the neutralizing antibodies [33]. Nowadays, genotypic classification of ARV strains is performed using RT-PCR in combination with phylogenic analysis or other molecular techniques, such as RFLP [7,26]. All phylogenic studies have classified ARV isolates into various groups and lineages; however, meta-analytically, there were no identical patterns. Such results suggested that different ARV genome segments may evolve in independent manner [27]. It is puzzling that no correlation was found between genotypes, serotypes and pathotypes, given detectable genetic differences of different ARV strains [7]. Divergences between genotypes, serotypes and pathogenicity suggested the involvement of multiple genes and proteins in serologic and pathogenic determination [33]. It may also result from high mutation rate of viral RNA and the possible reassortment between 2 strains co-infecting the same host [27,39]. Geographic characteristic conditions could facilitate exchanges between segmented genomes within small numbers of viral strains, which would result in less genetic variation within a region [33].

Vaccination against reovirus infections generally involves a live S1133 vaccine followed by an inactivated vaccine containing strains 1733 and/or 2408, knowing to be related to tenosynovitis and stunting syndrome pathologic conditions and belonging to the same subtype [40,41]. Although chickens were vaccinated, ARV pathogens still persist in Tunisian poultry. Serotyping studies are therefore needed to further elucidate the antigenic characteristics of these isolates and adapt the vaccination program.

## Conclusion

In the present study, we characterize genetically avian reovirus Tunisian strains isolated from commercial chickens reared in different geographic areas, during a 12 year period. The classification of ARV could be realized using the σC gene, the most variable known gene but other gene studies may provide additional information. It was not possible, however, to correlate the genotype of isolated strains with neither a specific pathologic conditions nor a geographic or temporal parameter. Although the vaccination against ARV is used in farms, the pathogens still persist in Tunisian poultry flocks. Serotyping studies are underway to characterize the antigenic proprieties of these isolates, which will help adapting the vaccination program.

## Abbreviations

AA: Amino acid; ARV: Avian reoviruses; SPF: Specific pathogen free; CEF: Chicken embryo fibroblast; CELC: Chicken embryo liver cells; CPE: Cytopathic effect; ARV: Avian reovirus; RT: Reverse transcription; N-PCR: Nested PCR; RSS: Runting-Stuning Syndrome; MAS: Malabsorption Syndrome; RFLP: Restriction fragment length polymorphism.

## Competing interests

The authors declare that they have no competing interests.

## Authors’ contributions

HKY is a PhD student, who carried out the avian reovirus genes detection by RT-PCR, was involved in sequencing and phylogenic studies and drafted the manuscript. BH and GL helped with experimental procedures and manuscript preparation. SAD reviewed the manuscript critically for important intellectual content. GA conceptualized the study, was involved in design of the trials, supervised all facets of the research and assisted in the writing process. All authors have red and approved the final manuscript.
